# The safety and efficacy of the re-administration of gilteritinib in a patient with FLT3-mutated R/R AML with CNS relapse: a case report

**DOI:** 10.3389/fonc.2024.1402970

**Published:** 2024-07-02

**Authors:** Yueru Ji, Zhuo Wan, Jian Yang, Miaowang Hao, Li Liu, Weiwei Qin

**Affiliations:** Department of Hematology, Second Affiliated Hospital of Air Force Military Medical University, Xi’an, Shaanxi, China

**Keywords:** AML, gilteritinib, CNS relapse, FLT3-ITD, target therapy

## Abstract

FLT3-ITD is a type of poor prognostic factors in acute myeloid leukemia (AML) disease. Gilteritinib, the second-generation FLT3 tyrosine kinase inhibitor, improved the overall survival of patients with relapsed/refractory FLT3-mutated AML in the ADMIRAL phase III trial. However, few data are available on the efficacy and safety of gilteritinib-based therapy for FLT3-mutated AML with central nervous system (CNS) involvement. We performed gilteritinib to treat a patient with CNS relapsed AML after allogeneic hematopoietic stem cell transplantation. The positive antileukemic effect of gilteritinib may bring new hope for the treatment of FLT3-mutated AML with CNS relapse.

## Introduction

Acute myeloid leukemia (AML) is a malignant disease of hematopoietic stem cells with clonal evolution of abnormally differentiated pluripotent stem/progenitor cells. The FMS-like tyrosine kinase 3 (FLT3) gene encodes a class III receptor tyrosine kinase and plays an important role in hematopoiesis. The internal tandem duplication of FLT3 (FLT3-ITD) is a poor prognostic factor in AML, especially in patients with AML who cannot undergo allogeneic HSCT (1). Gilteritinib, a selective FLT3 inhibitor against both FLT3-ITD and point mutations in the tyrosine kinase domain (FLT3-TKD), significantly improved the poor outcome of patients with FLT3 mutations (2, 3). However, little has been reported about its efficacy after CNS relapse in AML so far. In this study, we analyzed the safety and efficacy of the re-administration of gilteritinib in a patient with FLT3-mutated relapsed/refractory (R/R) AML with central nervous system (CNS) relapse soon after bridging HSCT with gilteritinib.

## Patient’s case

**Figure 1:**
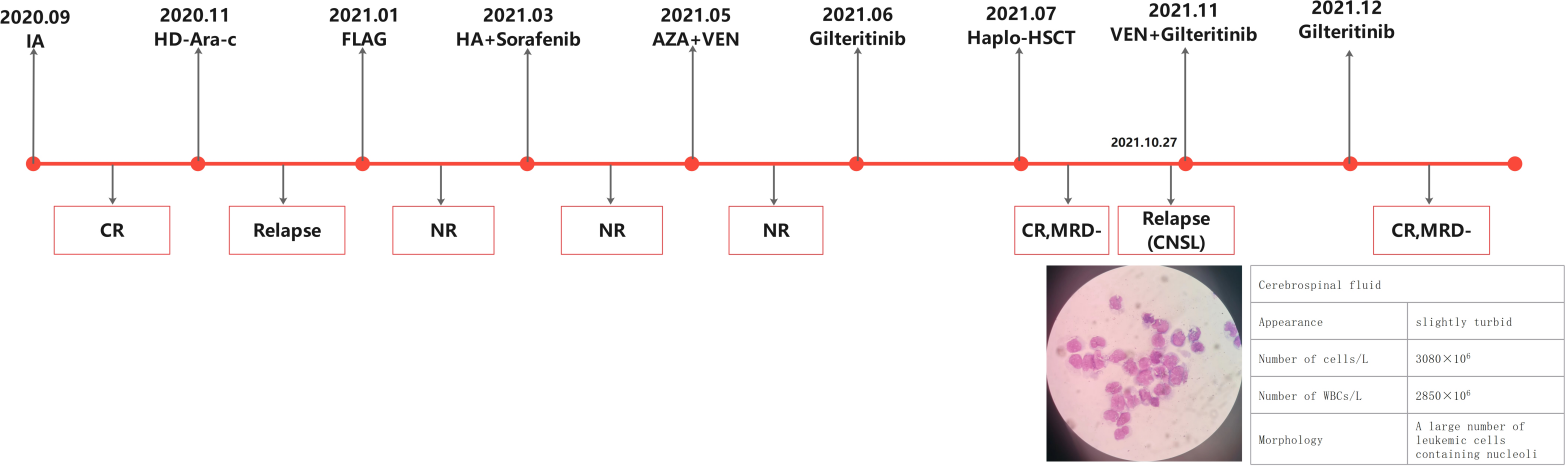
Previous chemotherapy regimens and treatment response.

A 39-year-old female presented to a hospital in September 2020 due to elevated white blood cell count and swollen gums and was initially diagnosed with AML (high-risk, FLT3-ITD mutation). First complete remission (CR) was achieved after induction therapy with idarubicin and cytarabine. However, the patient relapsed during consolidation phase with high-dose cytarabine (HD-Ara-c). After the failure of multiple lines of prior therapy involving sorafenib (**Figure 1**), the patient came to our department to seek further treatment. R/R FLT3-ITD-mutated (FLT3-ITD mutation frequency 30.6%, AR 0.388) AML was confirmed, and the patient received venetoclax (VEN, 200 mg/day) combined with azacitidine (AZA, 100 mg/day, D1–7) on May 11, 2021. Hematopoietic-related indicators (WBC 46.34 × 10E9/L, PLT 6 × 10E9/L, HGB 65 g/L) was elevated after 20 days of therapy. The bone marrow (BM) morphology showed myelodysplasia with 80.5% blasts, while an increased WBC count with 77% blasts was presented in the peripheral blood, which indicates another treatment failure. Since the patient had poor tolerance to chemotherapy and concurrent bilateral pneumonitis, she received gilteritinib therapy at 120 mg/day orally on June 8, 2021. At 15 days later, the BM examination revealed no blast cells, and measurable residual disease (MRD) was negative in the flow cytometry (FC) analysis. Haploidentical HSCT was performed on July 15, 2021, the conditioning regimen was CBA + ATG, and lumbar puncture sheath was given for prophylaxis of CNS leukemia after hematopoietic recovery from transplantation. On August 20, 2021, BM-CR was achieved with FC-MRD negative and FLT3-ITD mutation negative. However, the patient experienced right side tinnitus with decreased vision on the right side after 2 months. The cranial CT and MRI results showed no significant space-occupying lesions. A blood examination revealed WBC 2.22 × 10^9^/L, PLT 72 × 10^9^/L, and HGB 117 g/L. The BM showed no blasts with active hyperplasia, and FC-MRD remained negative. Cerebrospinal fluid (CSF) cytology after lumbar puncture showed multiple primitive cells that were visible on full radiograph, indicating the CNS relapse of AML. On November 4, 2021, cyclosporine as immunosuppressant was gradually reduced and finally stopped. In the meantime, VEN (200 mg/day) and gilteritinib (120 mg/day) were administered with twice-weekly intrathecal (IT) chemotherapy (cytarabine 50 mg + methotrexate 15 mg + dexamethasone 5 mg). An additional four sessions of IT injections (weekly) were given after CSF blast clearance. The drops in WBC (1.8 × 10^9^/L) and platelet (28 × 10^9^/L) occurred after 3 weeks, so the administration of VEN was discontinued with a reduced dose of gilteritinib (80 mg/day). BM remained CR with negative FC-MRD. In July 2022, the liver test showed hepatic injury with increased levels of ALT (754 U/L), AST (835 U/L), and TBIL (53 μmol/L). Oral polyene phosphocholine combined with diammonium glycyrrhizinate had a limited effect, and the patient was considered to have immune hepatic injury (liver GVHD or immune hepatic injury from gilteritinib). Oral methylprednisolone was started at 1 mg/kg for 7 days and was given in decreasing doses, while the patient continuously received gilteritinib with no dose adjustment. The results of a retest showed the improved level of AST (98 U/L) with the recovery of ALT and bilirubin levels. In September 2022, the patient’s CBC indicated that the neutrophils, platelets, and hemoglobin had returned to normal levels. As a result, we adjusted the dosage of gilteritinib to 120 mg/day. However, at 1 week later, the CBC result showed that the neutrophil count was 1.8 × 10^9^/L and the platelet count was 67 × 10^9^/L. The decision was made to continue gilteritinib at a dose of 80 mg/day for continuous treatment. By the time of the last follow-up on March 17, 2024, the CBC result indicated that the neutrophil count was 2.15 × 10^9^/L, the platelets were 127 × 10^9^/L, and hemoglobin was at 147 g/L. The bone marrow morphology showed remission, with negative flow MRD and no detectable FLT3-ITD mutation.

## Discussion

AML is a malignancy originating from hematopoietic stem cells and characterized by abnormal proliferation and differentiation of bone marrow blasts with clinical manifestations of cytopenias and transfusion dependence. The incidence of CNS involvement in AML varies between 1.1% and 32%, only sporadically at diagnosis and relapse. The prognosis is dismal, and most clinical trials have excluded patients with CNS-AML (4). A retrospective study showed that a greater proportion of monocytes (>40%) and elevated LDH and WBC (approximately 40 × 10^9^/L) were strong risk factors for CSF AML, as were patients with core binding factor and FLT3 mutations (5).

FLT3 mutations have been identified in approximately 30% of AML patients and will activate the intracellular complex in the kinase signaling pathway, which, in turn, promotes AML cell proliferation and inhibits differentiation and apoptosis (6). FLT3-mutated AML is associated with a higher risk of relapse and shorter survival than the wild-type (WT) FLT3 (7). Gilteritinib, a targeted agent developed by Astellas, Japan, for the treatment of FLT3-mutated AML, shows a significant inhibitory activity against FLT3-ITD and FLT3-TKD mutations (8–10). In 2019, the phase 3 ADMIRAL trial demonstrated the efficiency of gilteritinib monotherapy for patients with FLT3-mutated R/R AML, significantly prolonging overall survival (OS) compared with four standard salvage chemotherapy regimens (median OS 9.3 vs. 5.6 m, HR 0.64, *P* < 0.001) (11). We would like to emphasize the importance of incorporating FLT3 inhibitors into the induction therapy for patients with FLT3 mutations, specifically FLT-ITD mutations, at the time of initial diagnosis, as it has a significant impact on patient prognosis. It is worth mentioning that the failure to include FLT3 inhibitors, such as gilteritinib, in combination with the “3 + 7” regimen at the time of initial diagnosis might be a contributing factor to the refractory nature observed in this particular patient.

Clinical data on gilteritinib treating FLT3-mutated AML with extramedullary (EM) involvement are lacking, and there are only several case reports to date. It is recommended in these studies that FLT3-mutated AML with EM recurrence should be treated with bridging therapy with gilteritinib followed by HSCT. It is worth noting that gilteritinib for EM relapse can be also effective after allogeneic HSCT (9, 11). Our R/R AML case remained refractory prior to treatment with sorafenib, multiple lines of intensive chemotherapy, and HSCT. Followed by gilteritinib bridging treatment, the patient finally achieved CR. Unfortunately, she experienced central relapse after 3 months. Considering that there is currently no clear evidence of gilteritinib concentration into the CSF, we administered venetoclax plus gilteritinib for 3 weeks. VEN was discontinued due to hematologic toxicity, and gilteritinib was fixed at 80 mg/day. For now, the patient still remains in remission. We decided against pursuing a second transplant after the patient achieved remission following the CNS relapse. Firstly, we took into consideration the satisfactory response to targeted therapy in treating the CNS involvement, along with the poor efficacy of secondary HSCT in CNS treatment, as well as the significant adverse effects associated with transplantation. Under these circumstances, we believed that a second transplant would not provide a survival benefit for the patient. Secondly, the patient expressed fear and reluctance toward undergoing allogeneic hematopoietic stem cell transplantation, which further influenced our decision not to proceed with a second HSCT.

In line with the previous reports, this case demonstrates the effectiveness of gilteritinib in treating CNS relapse, although we failed to detect the blood concentration of gilteritinib in the CSF. Meanwhile, we also draw the following lessons from this case: (1) early maintenance therapy with gilteritinib after bridging allogeneic HSCT for refractory FLT3-mutated AML (successful engraftment and resume the therapy as soon as possible) and (2) allogeneic HSCT still carries a high risk of CNS relapse, especially in patients with FLT3 mutations, so risk-tailored prophylaxis is suggested. In conclusion, gilteritinib, as a bridge regimen to transplant in R/R FLT3-mutated AML and maintenance for isolated CNS relapse after HSCT, is safe and effective. An early resume of gilteritinib as soon as possible after hematopoietic recovery following transplantation is recommended to achieve the goal of preventing CNS and/or BM relapse.

## Data availability statement

The original contributions presented in the study are included in the article/supplementary material. Further inquiries can be directed to the corresponding author.

## Ethics statement

Written informed consent was obtained from the individual(s) for the publication of any potentially identifiable images or data included in this article.

## Author contributions

YJ: Methodology, Writing – original draft. ZW: Validation, Writing – review & editing. JY: Data curation, Writing – review & editing. MH: Investigation, Writing – review & editing. LL: Resources, Writing – review & editing. WQ: Conceptualization, Writing – original draft.
